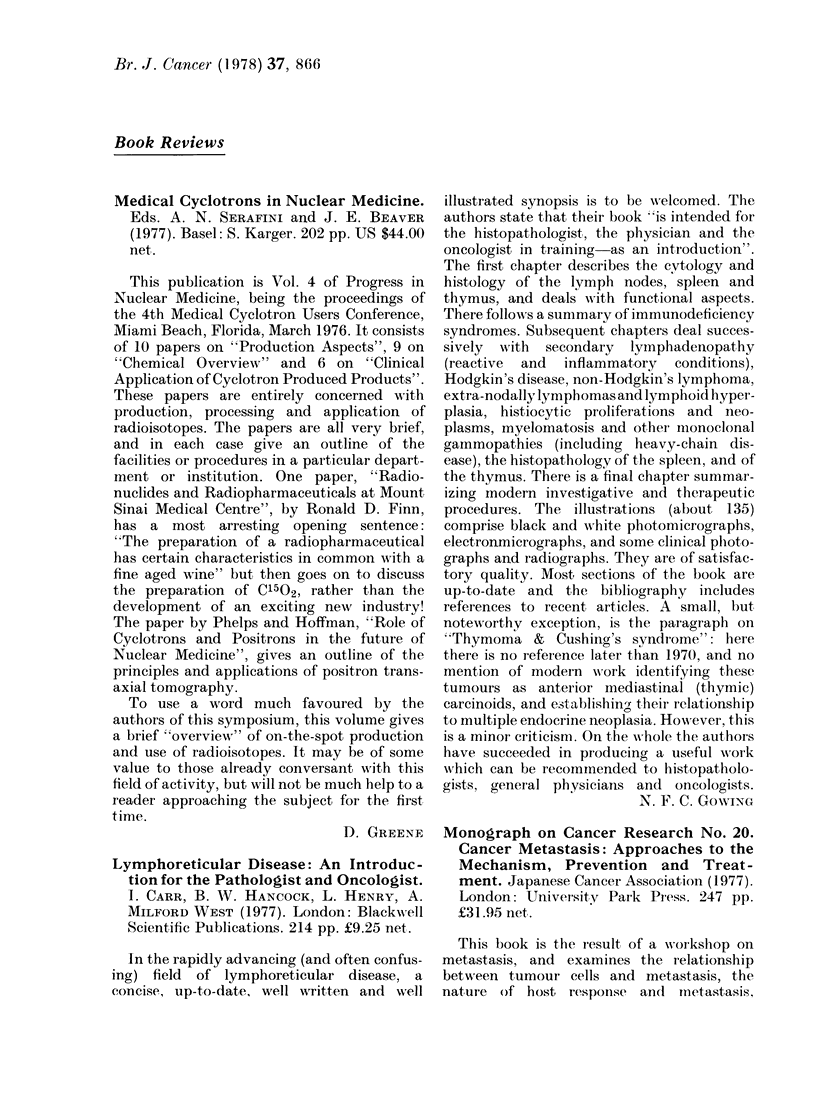# Lymphoreticular Disease: An Introduction for the Pathologist and Oncologist

**Published:** 1978-05

**Authors:** N. F. C. Gowing


**Lymphoreticular Disease: An Introduction for the Pathologist and Oncologist.** I. CARR, B. W. HANCOCK, L. HENRY, A. MILFORD WEST (1977). London: Blackwell Scientific Publications. 214 pp. £9.25 net.

In the rapidly advancing (and often confusing) field of lymphoreticular disease, a concise, up-to-date, well written and well illustrated synopsis is to be welcomed. The authors state that their book "is intended for the histopathologist, the physician and the oncologist in training—as an introduction". The first chapter describes the cytology and histology of the lymph nodes, spleen and thymus, and deals with functional aspects. There follows a summary of immunodeficiency syndromes. Subsequent chapters deal successively with secondary lymphadenopathy (reactive and inflammatory conditions), Hodgkin's disease, non-Hodgkin's lymphoma, extra-nodally lymphomas and lymphoid hyperplasia, histiocytic proliferations and neoplasms, myelomatosis and other monoclonal gammopathies (including heavy-chain disease), the histopathology of the spleen, and of the thymus. There is a final chapter summarizing modern investigative and therapeutic procedures. The illustrations (about 135) comprise black and white photomicrographs, electronmicrographs, and some clinical photographs and radiographs. They are of satisfactory quality. Most sections of the book are up-to-date and the bibliography includes references to recent articles. A small, but noteworthy exception, is the paragraph on "Thymoma & Cushing's syndrome": here there is no reference later than 1970, and no mention of modern work identifying these tumours as anterior mediastinal (thymic) carcinoids, and establishing their relationship to multiple endocrine neoplasia. However, this is a minor criticism. On the whole the authors have succeeded in producing a useful work which can be recommended to histopathologists, general physicians and oncologists.

N. F. C. GOWING